# Vaccine Literacy and Vaccination: A Systematic Review

**DOI:** 10.3389/ijph.2023.1605606

**Published:** 2023-02-14

**Authors:** Enming Zhang, Zhengyue Dai, Suxing Wang, Xiaolong Wang, Xian Zhang, Qiong Fang

**Affiliations:** ^1^ School of Nursing, Shanghai Jiao Tong University, Shanghai, China; ^2^ Nursing Department, Caohejing Community Health Service Center, Shanghai, China

**Keywords:** health literacy, vaccine hesitancy, vaccination, vaccination attitude, vaccine literacy

## Abstract

**Objectives:** Vaccine literacy (VL) is an essential component of health literacy and is regarded as the promising technique for eliminating vaccine hesitancy. This review summarizes the relationship between VL and vaccination, including vaccine hesitancy, vaccination attitude, vaccination intention, and vaccination uptake.

**Methods:** A systematic search was conducted in the PubMed, Embase, Web of Science, CINAHL, PsycINFO, and Cochrane Library databases. Studies that explored the relationship between VL and vaccination were included, and the PRISMA recommendations were followed.

**Results:** 1523 studies were found, and 21 articles were selected. The earliest article was published in 2015 and focused on the HPV vaccination and VL of female college students. Three studies surveyed parents’ VL about childhood vaccinations, and the remaining 17 focused on COVID-19 VL in different groups.

**Conclusion:** Although VL plays a role in determining the level of vaccine hesitancy across various populations, the association remains unclear. In the future, additional assessment methods could be developed and used to conduct prospective cohort and longitudinal studies to determine the causal relationship between VL and vaccination.

## Introduction

Vaccination is currently one of the most effective and cost-efficient public health interventions in the world (1). According to the World Health Organization (WHO), vaccination can prevent between two and three million deaths annually across the globe (2). Non-etheless, in many regions of the world, vaccination continues to be questioned and rejected (3). Vaccine hesitancy is the delay or refusal of vaccination despite the availability of immunization services. It varies by time, region, and vaccination type, based on complicated and specific circumstances (4). Although under-vaccination is caused by many complex factors related to access or pragmatism, vaccine hesitancy is still considered a major cause of declining vaccination rates and outbreaks of vaccine-preventable diseases (5, 6). In 2015–2017, more than 90 percent of the 194 WHO member states reported vaccine hesitancy (7). In 2019, the WHO listed vaccine hesitancy as one of the top ten global health challenges (2), given the severity and prevalence of the problem. To combat COVID-19 and other vaccine-preventable diseases more effectively, it is necessary to address and eliminate vaccine hesitancy (8, 9).

To maintain or improve one’s quality of life over the course of a lifetime, one must have a high level of health literacy (HL), which is described as the knowledge, motivation, and capacity to find, comprehend, assess, and apply relevant health information to one’s circumstances (10). The relationship between HL and vaccination, including attitudes toward vaccines, vaccination intentions, and vaccine uptake, was thoroughly and systematically evaluated. It was discovered that HL may be influenced by crucial factors like country, age, and vaccine type in predicting acceptance or resistance to vaccines (11). However, more research is needed to understand the importance of HL in predicting vaccination.

When examining WHO’s global vaccination goals and strategies in March 2011, Ratzan SC, a Harvard scholar, first introduced the concept of VL, which states that VL is the ability of people to access, process, and understand basic vaccination knowledge and vaccination services, as well as to assess the potential consequences and risks of their behavior and make health-related decisions (3). This definition encompasses the functional, interactive/communicative, and essential characteristics of VL. From a psychometric perspective, functional VL questions focus more on linguistic ability and involve semantic systems, whereas interactive/communicative questions focus more on cognitive effort, such as problem solving and decision making (10, 12, 13). VL is based on the same concept of HL and has been identified as one of the defining attributes of VL in a conceptual analysis study by Filipino scholars (14). The globalized COVID-19 epidemic is an ideal opportunity to enhance the resistance to wrong information about vaccination and promote vaccination (15). VL is likely to be the promising tool for overcoming vaccine hesitancy because it manages information transfer and facilitates dialog (16–18). Although vaccine hesitancy and vaccine literacy are not the only determinants of vaccination coverage, improving the public’s vaccine literacy and reducing their vaccine hesitancy are very relevant to promote vaccination and immunization planning efforts.

Existing VL measurement tools are limited. Ishikawa et al. (19) developed a health literacy scale for diabetic patients based on Nutbeam’s health literacy model (12) in 2008. The scale has been used consistently in clinical and preventative medicine. Health Literacy about Vaccination of Adults in Italian (HLVa-IT) was created by Biasio et al. (13) based on Ishikawa, primarily to assess adult vaccination health literacy in Italy. It is also currently the most mature and most used tool (20–22). There were 14 items in total, including functional and interactive-critical items in two dimensions. Aharon’s Vaccine Health Literacy Scale (23) was also based on an adaptation of Ishikawa, to assess the vaccine health literacy of parents of young children. It comprised thirteen items and three dimensions: functional, communicative, and critical. There are also studies that have used VL measured by self-report questionnaires (24, 25).

This systematic review aims to present the latest evidence on the relationship between VL and vaccination, including vaccine hesitancy, vaccination attitudes, vaccination intentions and vaccine uptake. It is hoped that this study will provide new perspectives and ideas for preventing or intervening in the occurrence of vaccine hesitancy and for advancing vaccination efforts to play a more prominent role in the prevention and control of infectious diseases.

## Methods

### Data Sources and Searches

A medical librarian skilled in systematic searching constructed a search using a combination of subject headings and keywords to represent the concepts of VL and vaccination. The databases MEDLINE *via* PubMed, Embase *via* Elsevier, Web of Science Core Collection Citation Indexes *via* Clarivate, Cumulative Index of Nursing and Allied Health Literature (CINAHL) *via* EBSCO, APA PsycINFO *via* EBSCO, and Cochrane Central Register of Controlled Trials (CENTRAL) *via* Cochrane Library were searched from their inception to 14 July 2022. The language of the literature is limited to English. Non-human research, editorials, opinions, and conference abstracts were eliminated whenever possible (see attached search strategy Supplementary Appendix Tables SA1–SA6).

### Study Selection and Data Extraction

Inclusion criteria included vaccine hesitancy, vaccination attitude, vaccination intention, and vaccine uptake from selected English-language primary studies on the relationship between VL and vaccination. This systematic review examined all types of vaccinations. We considered research on individuals of all ages whose VL was measured using an instrument that assessed functional VL, interactive or communicative VL, and critical VL. Excluded were papers that reported solely on vaccination literacy or VL scores.

In this literature assessment, we first eliminated duplicates originating from different databases. Then, titles and abstracts were evaluated to assess eligibility, followed by full-text reviews by at least two researchers using the Covidence tool. To prevent omissions, we verified all references included in the recognized literature. With the help of a third researcher, disagreements regarding inclusion or exclusion were settled through consensus.

### Quality Assessment

All included studies’ methodological quality was evaluated using the STROBE declaration established by von Elm et al. (26). Totaling 22 items, the checklist evaluates the quality of observational research in terms of title and abstract, introduction, methods, results, and discussion. Two reviewers assessed the methodological quality of all included studies separately. During a consensus conference, any conflicts between the two reviewers regarding quality ratings were settled; a third reviewer was consulted in case of a discrepancy. Each criterion was evaluated with a score of 1 (yes) or 0 (no), giving each bar equal weight. A total quality score was awarded to each study by aggregating the scores obtained for each item. This systematic review is registered with PROSPERO (# CRD42022346197).

## Results

### Literature Search

**FIGURE 1:**
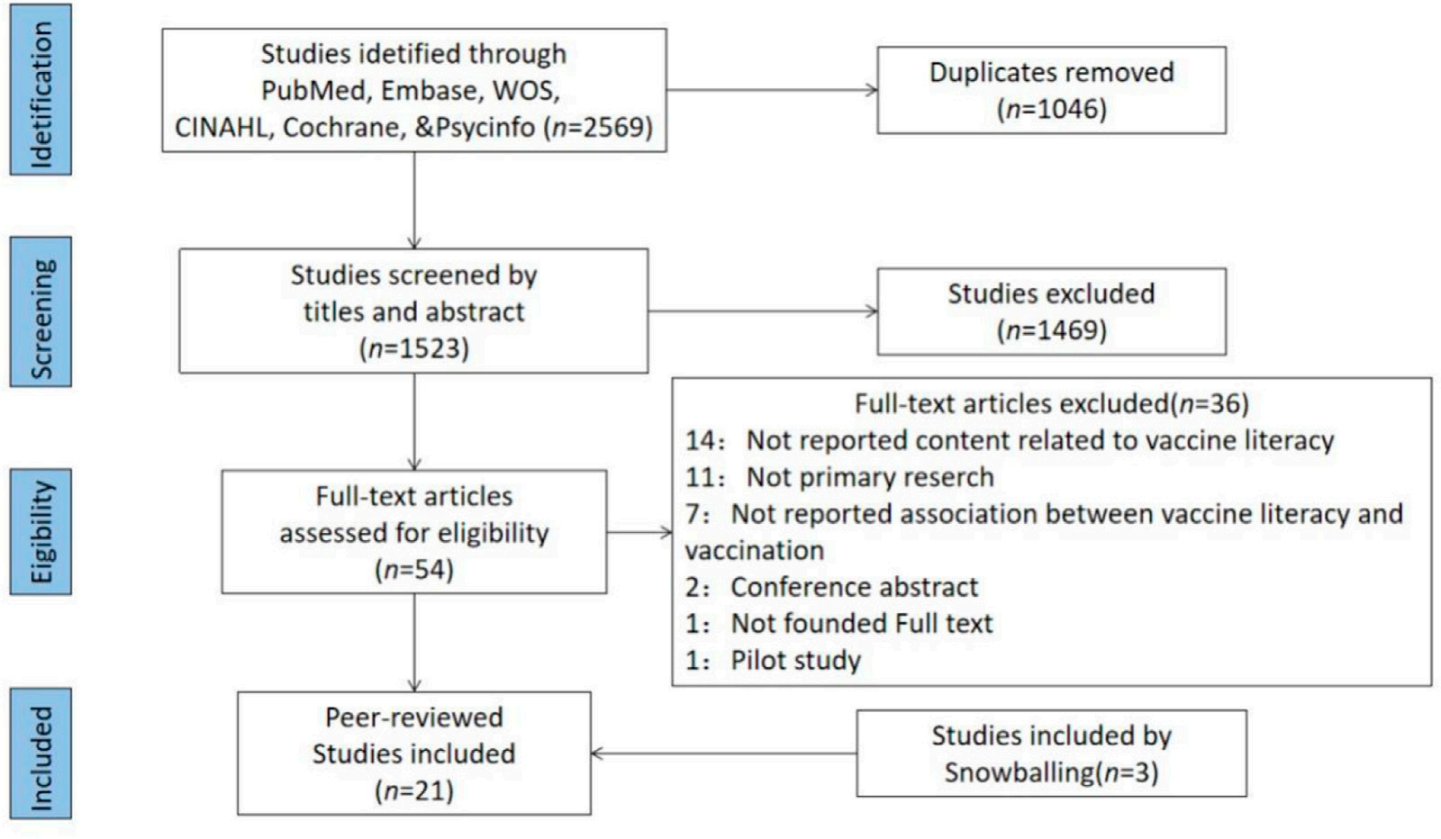
PRISMA Flow Diagram of study search and selection (Shanghai, China, 2022).

Figure 1 illustrates the processes we used to filter the literature using the PRISMA checklist (27). Our search method returned 1,523 studies, of which only 21 matched our review requirements. Supplementray Appendix Table SA7 lists the main features of all the studies included in the systematic review. All the studies were observational. They were mainly based on a cross-sectional design, with one study using a retrospective telephone follow-up survey, three studies using a paper-based questionnaire face-to-face, and the remainder using an online questionnaire.

In addition, although articles reporting only VL scores were not included, because some studies were useful for this systematic review, these papers are discussed in our discussion section.

### General Characteristics

The earliest article was published in 2015 and focused mainly on the HPV vaccination and VL of female college students at a public university in the Midwestern United States. Three studies surveyed parents’ VL about hepatitis B, diphtheria, tetanus, pertussis, mumps, measles, rubella, and dengue vaccinations to promote children’s immunization. Previously, the global epidemic of COVID-19 continues to expand, and how to increase the coverage of the COVID-19 vaccination for the entire population has been the focus of academics worldwide. So, research on COVID-19 VL has grown a lot among adults, parents of young children, college students, doctors, and other groups. Of these, eight studies were conducted in low- and middle-income countries (LMICs), and 13 studies were conducted in high-income developed countries.

### VL and Vaccination Among College Students

VL is a significant issue influencing the vaccination decisions of college students. A study of female college students in the United States revealed that HPV VL is a strong predictor of HPV vaccine completion (24). A study in Saudi Arabia (28) found that nursing students who planned to vaccinate against COVID-19 had high levels of interactive-critical COVID-19 VL.

### Children’s Parents’ VL and Children’s Vaccination

Several studies on the VL of children’s parents and vaccination of children have yielded inconclusive results. According to an Israeli study, the connection between communicative VL and vaccine compliance is strongly adverse; parents with highly functional, communicative, and critical VL are more probably not to vaccinate their children (23). The outcomes of the other three studies were contrary to this one. Following erroneous rumors of a locally manufactured kid vaccine scandal, a Chinese study analyzes the correlation between VL and vaccination-related consequences (25). It was discovered that parents with higher functional and critical VL were more likely to choose the domestic vaccine. One study examined the association between VL and maternal uptake of the dengue vaccine in children (29). Mothers’ functional and critical VL levels exhibited a significant correlation with vaccination acceptance. But the mothers’ willingness to vaccinate had nothing to do with their VL score. Parents who intend to vaccinate their children with the COVID-19 vaccine showed more significant levels of functional, interactive, and critical skills and total VL, a more favorable impression of the vaccine, and less reported vaccination reluctance than parents who do not intend to vaccinate their children (30).

### Adults’ VL and Vaccination

Seven studies have examined the relationship between adult VL and COVID-19 vaccination results (20, 22, 31–35). Of these, only one study conducted in Bangladesh found no association between vaccination literacy and intention to receive the COVID-19 vaccine, in contrast to the other 6 studies (32). Three studies have examined the connection between adult VL and hesitation to receive a booster dose of COVID-19 vaccination (36–38). The VL for functional, interactive, communicative, and critical VL were all much greater in the group that would get boosters than in the group that would not.

### Patient’s VL and Vaccination

Accepting the COVID-19 vaccination was strongly associated with the interactive VL of cancer patients (39). However, no significant correlation was identified between the willingness to receive the vaccine and the VL function. VL is associated with supportive views and attitudes of the COVID-19 vaccine but not with current vaccination behavior, where interactive critical VL was more likely to be associated with positive beliefs about the COVID-19 vaccination, according to a survey of patients with systemic autoimmune diseases aged 18 and older in Khairi (40).

### Healthcare Professionals’ VL and Vaccination

A study conducted at the only public tertiary care hospital in Barbados found that healthcare professionals (HCPs) who were willing to receive the COVID-19 vaccine had lower VL levels than those who were unwilling to get vaccinated promptly (41). In addition, this study revealed that HCPs with a higher VL viewed the COVID-19 immunization as safe and would suggest it to a friend if given a chance.

### VL and Vaccination Among Health Volunteers

The association between VL and increased COVID-19 vaccination among Thailand’s village health volunteers (VHVs) was not statistically significant (42). However, the study also evaluated the preferences of VHVs for COVID-19 vaccine types. The findings indicated that VL substantially reversed the acceptance of the COVID-19 vaccine among VHVs who chose the mRNA vaccine.

### VL and Vaccination Among Young Women

The intention to receive the COVID-19 vaccine and VL among younger Australian women residing in rural and remote areas were not significantly correlated (43). Nevertheless, this study’s qualitative interviews revealed that two critical factors influenced young rural women’s vaccination: their assessment that the evidence on the COVID-19 vaccine and infection was inadequate for making vaccination decisions, resulting in widespread confusion and uncertainty, and their feeling of being overwhelmed or confused by information.

### Quality Assessment Summary

The quality assessment scores of 21 selected articles are listed in Supplementary Appendix Table SA8. When evaluating articles, the two researchers had varying opinions, primarily about biases, quantitative variables, and other analyses, typically resulting from short descriptions or incorrect explanations. All initial disagreements were settled through consensus-building meetings.

All studies scored higher than 60% (14.4), with scores ranging between 15/24 (62.5%) and 21/24 (87.5%). Although the study by Krishnamurthy et al. received the lowest score, the results and discussion of the study provided a thorough and structured analysis and met our inclusion criteria. Therefore, we have included this article (41).

All studies contain thorough information in the title, abstract, and introduction, as well as an indication of the study type. In addition, they evaluate the generalizability of the findings and present a careful assessment of the results’ overall significance. Only two research reports addressed potential causes of bias, such as the inclusion of control variables, methods to prevent respondents from responding more than once, and the exclusion of surveys that were finished more rapidly (32, 36). Only five researchers conducted additional statistical analyses, including path analysis and others (23, 30, 32, 38, 42).

## Discussion

The concept of vaccination literacy is still relatively novel, which is one of the factors contributing to the global increase in vaccine hesitancy. This systematic review provides an updated synthesis of the available information on the role of VL in predicting vaccine hesitancy and other vaccination-related outcomes and may offer significant recommendations for the post-pandemic period of COVID-19.

### Measurement of VL

Given that vaccination attempts to prevent infectious diseases in both the individual and the population (herd immunity), VL’s relevance in disease prevention is obvious (16). It is crucial to note that there are now only a few measurement tools for vaccination literacy. Regardless, most of the studies examined in this analysis used psychometrically assessed measures to measure VL. However, it is crucial to note that the scales employed in these studies were modified versions of those used to measure health literacy in people with diabetes.

Although the link between VL and health literacy is obvious, the United Nations has recognized VL as a crucial element in guaranteeing the growth of health literacy (3). However, some research has found a clear correlation between health literacy abilities and vaccine acceptability, while others have found a negative correlation or none. It may be influenced by different factors, including different population settings, the type of vaccine being evaluated, and the characteristics of the test used (44). These observations suggest why more consideration should be given to further broadening the way VL is measured rather than just adapting health literacy assessment tools.

In addition, as vaccination literacy has progressed, measures for testing VL have been modified throughout time, particularly for the VL of the COVID-19 vaccination. However, current VL assessments have been employed inconsistently to measure VL in public. Measuring high levels of VL in the presence of information overload may potentially result in information evaluation errors (44). In addition, current VL measurement tools are still derived from health literacy measurement tools, and specific dimensions of VL are under-measured. This is despite the progressive improvement in the understanding of VL and the number of studies in numerous countries. Therefore, measurement methodologies for VL must be strengthened by incorporating vaccination context and specialized knowledge components. Cultural differences between countries or regions should also be considered when analyzing and quantifying VL in the future.

### The Complexity of Factors Influencing VL

The improvement of VL in the entire population helps the public recognize the value of vaccination, increases trust in vaccination, and contributes to the immunization of the whole population with vaccines (3). Understanding the factors determining vaccination literacy is crucial for more successful intervention implementation.

VL may be affected by demographic factors such as gender, age, and socioeconomic level. Women have more robust levels of VL than men (40), and another study similarly found that women had less difficulty accessing and understanding information about vaccine scandals (25). There is some disagreement about age, with one study claiming that VL decreases with age (20), while Cadeddu et al. (45) discovered that VL increases with age group. Education level correlates positively with vaccination literacy (20), and research by Correa-Rodrguez et al. (22) found significant inequalities in VL among individuals with varying levels of education. In addition, lower economic poverty and higher socioeconomic status were associated with higher VL levels of VL (40, 45). There was also a link between interactive-critical VL and the area of residence, citizenship, and socioeconomic status (40).

As a way to improve health, vaccination is inextricably linked to a person’s health status, which is affected by several personal factors. A survey of adults in Italy discovered that (45), regardless of the frequency of people’s visits, a person’s self-rated health status, the presence of a chronic disease or other health problem, any limitations imposed by that problem, and access to professional healthcare training were all associated with vaccine knowledge. VL may also influence people’s utilization of critical public health services.

Socioculture can have an impact on individuals’ behavioral intentions to vaccinate, which in turn can influence the spread of infectious diseases. Different sociocultural characteristics may have contributed to considerably lower levels of HPV vaccine knowledge among Asian American and Pacific Islander (AAPI) students than non-Latino White (NLW) students, according to Lee et al. (24). One study indicated that due to sociocultural shame or modesty norms, AAPI women were less likely to talk about or learn about cervical cancer prevention efforts (46).

Furthermore, Gusar et al. (3) discovered that participants with employment, chronic medical issues, medication use, or alcohol consumption had lower levels of VL. According to Rauh et al. (46), interpersonal ties, information sources, infrastructure, and policies may all be associated with VL. A survey of nursing home workers showed that people whose primary sources of information were government vaccination campaigns, general practitioners, or other health experts, and search engines had much higher total VL scores (47).

### VL in Low- and Middle-Income Countries (LMICs)

The WHO Vaccination Agenda 2030 seeks to adapt new technologies to the persistent challenges of infectious diseases, emphasizing vaccination as an investment in a healthier, safer, and more prosperous society (48). However, inequities in access to immunization within nations continue, and there remain difficulties in achieving goal vaccination rates (49). LMICs accounted for less than 40% of the studies we included (22, 28, 29, 32, 33, 35, 37, 39), more research in LMICs is required due to the importance of VL in vaccination. To prepare for the future’s unpredictability, countries could develop plans to increase vaccination rates and improve the public’s VL.

### Perspectives on the Use of VL in Health Education

The rising dissemination of disinformation *via* social media platforms and other means poses a substantial risk to public health and places new demands on vaccination and health education activities (50). According to Michel and Goldberg (51), VL begins with education and can result in vaccination empowerment, increased vaccination confidence, faith in the healthcare system, and acceptance of vaccines. VL is disseminating professional vaccination information required by the public for efficient immunization planning in a manner that is as simple to comprehend as possible. It also necessitates immunization practitioners, and managers acquire a broader and deeper understanding of vaccines. Vaccination health education in the late stages of a pandemic can help people learn more about vaccines and fully mobilize the initiative and incentives for vaccination to help prevent and control infectious diseases in a more significant way.

### Limitation

This study includes as many relevant studies as feasible based on a comprehensive and methodical search strategy; however, even with snowball searches and peer recommendations from other papers; the literature search was mainly database based and did not include evidence searches of online research platforms, authoritative government reports or other non-traditional sources, which may introduce publication bias and selective reporting bias; in addition, only English literature was selected and literature in other languages was not considered. Only studies that included VL in their research objectives or questions were included in this review. Therefore, this does not address the complete spectrum of interactions between VL and vaccination but concentrates on those studies that made VL a primary research focus. Therefore, conclusions obtained from this analysis should be limited to the measuring methods and breadth of these studies rather than a general assessment of the impact of VL on vaccination.

### Conclusion

Overall, although VL plays a role in determining the degree of vaccine hesitancy in different populations, this relationship remains unclear. VL is an important “endogenous driver” of people’s vaccine choices, overcoming vaccine hesitancy and increasing vaccination rates. However, the assessment tools in the literature included in this review were more limited, the influences examined were more complex, and the distribution across countries and regions was uneven. In the future, more specific assessment methods could be developed for prospective cohort and longitudinal studies to determine the causal relationship between VL and vaccination, thus providing a better basis for vaccine health education campaigns.

## References

[1] Robbins MJ Jacobson SH. Analytics for Vaccine Economics and Pricing: Insights and Observations. Expert Rev Vaccin (2015) 14(4):605–16. 10.1586/14760584.2015.985662 25435003

[2] WHO. Ten Threats to Global Health in 2019 [Internet] (2019). Available from: https://www.who.int/news-room/spotlight/ten-threats-to-global-health-in-2019 (Accessed February 06, 2023).

[3] Ratzan SC. Vaccine Literacy: A New Shot for Advancing Health. J Health Commun (2011) 16:227–9. 10.1080/10810730.2011.561726 21391044

[4] MacDonald NE, SAGE Working Group on Vaccine Hesitancy. Vaccine Hesitancy: Definition, Scope and Determinants. Vaccine (2015) 33(34):4161–4. 10.1016/j.vaccine.2015.04.036 25896383

[5] Dubé E Laberge C Guay M Bramadat P Roy R Bettinger J. Vaccine Hesitancy: an Overview. Hum Vaccin Immunother (2013) 9(8):1763–73. 10.4161/hv.24657 23584253 PMC3906279

[6] Bedford H Attwell K Danchin M Marshall H Corben P Leask J. Vaccine Hesitancy, Refusal and Access Barriers: The Need for Clarity in Terminology. Vaccine (2018) 36(44):6556–8. 10.1016/j.vaccine.2017.08.004 28830694

[7] Lane S MacDonald NE Marti M Dumolard L. Vaccine Hesitancy Around the globe: Analysis of Three Years of WHO/UNICEF Joint Reporting Form Data-2015-2017. Vaccine (2018) 36(26):3861–7. 10.1016/j.vaccine.2018.03.063 29605516 PMC5999354

[8] Ryan J Malinga T. Interventions for Vaccine Hesitancy. Curr Opin Immunol (2021) 71:89–91. 10.1016/j.coi.2021.05.003 34271335

[9] Rutten L Zhu X Leppin AL Ridgeway JL Jacobson RM. Evidence-Based Strategies for Clinical Organizations to Address COVID-19 Vaccine Hesitancy. Mayo Clinic Proc (2020) 96(3). 10.1016/j.mayocp.2020.12.024 PMC7772995 33673921

[10] Sørensen K Broucke SVD Fullam J Doyle G Pelikan J Slonska Z Health Literacy and Public Health: A Systematic Review and Integration of Definitions and Models. BMC Public Health (2012) 12(1):80–13. 10.1186/1471-2458-12-80 22276600 PMC3292515

[11] Lorini C Santomauro F Donzellini M Capecchi L Bechini A Boccalini S Health Literacy and Vaccination: A Systematic Review. Hum Vaccin Immunother (2018) 14(2):478–88. 10.1080/21645515.2017.1392423 29048987 PMC5806657

[12] Nutbeam D. Health Literacy as a Public Health Goal: a challenge for Contemporary Health Education and Communication Strategies into the 21st century. Health Promot Int (2000) 15(3):259–67. 10.1093/heapro/15.3.259

[13] Biasio LR Giambi C Fadda G Lorini C D’Ancona F D'Ancona F. Validation of an Italian Tool to Assess Vaccine Literacy in Adulthood Vaccination: A Pilot Study. Annali di Igiene: Medicina Preventiva e di Comunità. (2020) 32(3):205–22. 10.7416/ai.2020.2344 32266359

[14] Badua AR Caraquel KJ Cruz M Narvaez RA. Vaccine Literacy: A Concept Analysis. Int J Ment Health Nurs (2022) 31(4):857–67. 10.1111/inm.12988 35289065 PMC9111838

[15] Vanderpool RC Gaysynsky A Sylvia Chou WY. Using a Global Pandemic as a Teachable Moment to Promote Vaccine Literacy and Build Resilience to Misinformation. Am J Public Health (2020) 110(S3):S284–5. 10.2105/AJPH.2020.305906 33001735 PMC7532329

[16] Biasio LR. Vaccine Literacy Is Undervalued. Hum Vaccin Immunother (2019) 15(11):2552–3. 10.1080/21645515.2019.1609850 31013184 PMC6930053

[17] Sirikalyanpaiboon M Ousirimaneechai K Phannajit J Pitisuttithum P Jantarabenjakul W Chaiteerakij R COVID-19 Vaccine Acceptance, Hesitancy, and Determinants Among Physicians in a university-based Teaching Hospital in Thailand. BMC Infect Dis (2021) 21:1174. 10.1186/s12879-021-06863-5 34809607 PMC8607407

[18] Turhan Z Dilcen HY Dolu İ. The Mediating Role of Health Literacy on the Relationship between Health Care System Distrust and Vaccine Hesitancy during COVID-19 Pandemic. Curr Psychol (2022) 41:8147–56. 10.1007/s12144-021-02105-8 34312580 PMC8295547

[19] Ishikawa H Takeuchi T Yano E. Measuring Functional, Communicative, and Critical Health Literacy Among Diabetic Patients. Diabetes Care (2008) 31(5):874–9. 10.2337/dc07-1932 18299446

[20] Gusar I Konjevoda S Babić G Hnatešen D Čebohin M Orlandini R Pre-Vaccination COVID-19 Vaccine Literacy in a Croatian Adult Population: A Cross-Sectional Study. IJERPH (2021) 18(13):7073. 10.3390/ijerph18137073 34281009 PMC8297136

[21] Can Gür G Altinbaş Y. Covid-19 Literacy Scale: Turkish Validity and Reliability Study. Clin Nurs Res (2022) 31(3):404–12. 10.1177/10547738211059879 34814763

[22] Maneesriwongul W Butsing N Visudtibhan PJ Leelacharas S Kittipimpanon K. Translation and Psychometric Testing of the Thai COVID-19 Vaccine Literacy Scale. Pac Rim Int J Nurs Res (2022) 26(1):179–90.

[23] Amit Aharon A Nehama H Rishpon S Baron-Epel O. Parents with High Levels of Communicative and Critical Health Literacy Are Less Likely to Vaccinate Their Children. Patient Education Couns (2017) 100(4):768–75. 10.1016/j.pec.2016.11.016 27914735

[24] Lee HY Kwon M Vang S DeWolfe J Kim NK Lee DK Disparities in Human Papillomavirus Vaccine Literacy and Vaccine Completion Among Asian American Pacific Islander Undergraduates: Implications for Cancer Health Equity. J Am Coll Health (2015) 63:316–23. 10.1080/07448481.2015.1031237 25836058

[25] Wang X Zhou X Leesa L Mantwill S. The Effect of Vaccine Literacy on Parental Trust and Intention to Vaccinate after a Major Vaccine Scandal. J Health Commun (2018) 23:413–21. 10.1080/10810730.2018.1455771 29589807

[26] von Elm E Altman DG Egger M Pocock SJ Gøtzsche PC Vandenbroucke JP. The Strengthening the Reporting of Observational Studies in Epidemiology (STROBE) Statement: Guidelines for Reporting Observational Studies. The Lancet (2007) 370(9596):1453–7. 10.1136/bmj.39335.541782.AD 18064739

[27] Moher D Liberati A Tetzlaff J Altman DG. The PRISMA Group. Preferred Reporting Items for Systematic Reviews and Meta-Analyses: The PRISMA Statement. Plos Med (2009) 6(7):e1000097. 10.1371/journal.pmed.1000097 19621072 PMC2707599

[28] Alshehry AS Cruz JP Alquwez N Alsharari AF Tork HMM Ju A Predictors of Nursing Students’ Intention to Receive COVID‐19 Vaccination: A Multi‐university Study in Saudi Arabia. J Adv Nurs (2022) 78:446–57. 10.1111/jan.15002 34363635 PMC8446957

[29] Sumile EF Diric JH Dorado ZM Dumaua K Ecura M Dumaya JM. Dengue Vaccine Controversy Awareness, Vaccine Health Literacy, and Vaccine Acceptability Among Mothers in Select Rural Communities. J Health Caring Sci (2020) 2(22):123–34. 10.37719/jhcs.2020.v2i2.oa005

[30] Gendler Y Ofri L. Investigating the Influence of Vaccine Literacy, Vaccine Perception and Vaccine Hesitancy on Israeli Parents’ Acceptance of the COVID-19 Vaccine for Their Children: A Cross-Sectional Study. Vaccines (2021) 9(12):1391. 10.3390/vaccines9121391 34960137 PMC8703688

[31] Biasio LR Bonaccorsi G Lorini C Pecorelli S. Assessing COVID-19 Vaccine Literacy: a Preliminary Online Survey. Hum Vaccin Immunother (2021) 17(5):1304–12. 10.1080/21645515.2020.1829315 33118868 PMC8078752

[32] Nath R Imtiaz A Nath SD Hasan E. Role of Vaccine Hesitancy, eHealth Literacy, and Vaccine Literacy in Young Adults’ COVID-19 Vaccine Uptake Intention in a Lower-Middle-Income Country. Vaccines (2021) 9(12):1405. 10.3390/vaccines9121405 34960151 PMC8704098

[33] Engelbrecht M Heunis C Kigozi G. COVID-19 Vaccine Hesitancy in South Africa: Lessons for Future Pandemics. IJERPH (2022) 19(11):6694. 10.3390/ijerph19116694 35682278 PMC9180246

[34] Engelbrecht MC Kigozi NG Heunis JC. Factors Associated with Limited Vaccine Literacy: Lessons Learnt from COVID-19. Vaccines (2022) 10(6):865. 10.3390/vaccines10060865 35746473 PMC9229188

[35] Omidvar S Firouzbakht M. Acceptance of COVID-19 Vaccine and Determinant Factors in the Iranian Population: a Web-Based Study. BMC Health Serv Res (2022) 22(1):652. 10.1186/s12913-022-07948-w 35578251 PMC9108146

[36] Yadete T Batra K Netski DM Antonio S Patros MJ Bester JC. Assessing Acceptability of COVID-19 Vaccine Booster Dose Among Adult Americans: A Cross-Sectional Study. Vaccines (2021) 9(12):1424. 10.3390/vaccines9121424 34960170 PMC8703732

[37] Achrekar GC Batra K Urankar Y Batra R Iqbal N Choudhury SA Assessing COVID-19 Booster Hesitancy and its Correlates: An Early Evidence from India. Vaccines (2022) 10((7):1048. 10.3390/vaccines10071048 35891212 PMC9323084

[38] Batra K Sharma M Dai CL Khubchandani J. COVID-19 Booster Vaccination Hesitancy in the United States: A Multi-Theory-Model (MTM)-Based National Assessment. Vaccines (2022) 10(5):758. 10.3390/vaccines10050758 35632514 PMC9144395

[39] khiari H Cherif I M’ghirbi F Mezlini A Hsairi M. COVID-19 Vaccination Acceptance and its Associated Factors Among Cancer Patients in Tunisia. Asian Pac J Cancer Prev (2021) 22:3499–506. 10.31557/APJCP.2021.22.11.3499 34837905 PMC9068194

[40] Correa-Rodríguez M Rueda-Medina B Callejas-Rubio JL Ríos-Fernández R de la Hera-Fernández J Ortego-Centeno N. COVID-19 Vaccine Literacy in Patients with Systemic Autoimmune Diseases. Curr Psychol (2022) 18:1–16. 10.1007/s12144-022-02713-y PMC8764502 35068910

[41] Krishnamurthy K Sobers N Kumar A Ojeh N Scott A Cave C COVID-19 Vaccine Intent Among Health Care Professionals of Queen Elizabeth Hospital. J Multidiscip Healthc (2021) 14:3309–19. 10.2147/JMDH.S336952 34876817 PMC8643144

[42] Siewchaisakul P Sarakarn P Nanthanangkul S Longkul J Boonchieng W Wungrath J. Role of Literacy, Fear and Hesitancy on Acceptance of COVID-19 Vaccine Among Village Health Volunteers in Thailand. PLoS ONE (2022) 17(6):e0270023. 10.1371/journal.pone.0270023 35749368 PMC9231694

[43] Carter J Rutherford S Borkoles E. COVID-19 Vaccine Uptake Among Younger Women in Rural Australia. Vaccines (2021) 10(1):26. 10.3390/vaccines10010026 35062687 PMC8778203

[44] Biasio LR Carducci A Fara GM Giammanco G Lopalco PL. Health Literacy, Emotionality, Scientific Evidence: Elements of an Effective Communication in Public Health. Hum Vaccin Immunother (2018) 14(6):1515–6. 10.1080/21645515.2018.1434382 29381399 PMC6037460

[45] Cadeddu C Regazzi L Bonaccorsi G Rosano A Unim B Griebler R The Determinants of Vaccine Literacy in the Italian Population: Results from the Health Literacy Survey 2019. Int J Environ Res Public Health (2022) 19(8):4429. 10.3390/ijerph19084429 35457297 PMC9029177

[46] Fang CY Ma GX Tan Y. Overcoming Barriers to Cervical Cancer Screening Among Asian American Women. N A J Med Sci (2011) 4(2):77–83. Fox Chase Cancer Center, Philadelphia, PA, Temple University, College of Health Professions, Center for Asian Health, Department of Public Health, Philadelphia, PA, Temple University, College of Health Professions, Center for Asian Health, Department of Public Health, Philadelphia, PA. 10.7156/v4i2p077 PMC3115728 21687826

[47] Lorini C Collini F Galletti G Ierardi F Forni S Gatteschi C Vaccine Literacy and Source of Information about Vaccination Among Staff of Nursing Homes: A Cross-Sectional Survey Conducted in Tuscany (Italy). Vaccines (2022) 10(5):682. 10.3390/vaccines10050682 35632438 PMC9144185

[48] World Health Organization. Immunization Agenda 2030: a Global Strategy to Leave No One behind. Available from: https://www.who.int/publications/m/item/immunization-agenda-2030-a-global-strategy-to-leave-no-one-behind (Accessed February 06, 2023). 10.1016/j.vaccine.2022.11.042 39004466

[49] World Health Organization, 2018 Assessment Report of the Global Vaccine Action Plan: Strategic Advisory Group of Experts on Immunization. World Health Organization; 2018.

[50] Zarocostas J. How to Fight an Infodemic. The Lancet (2020) 395(10225):676. 10.1016/S0140-6736(20)30461-X PMC7133615 32113495

[51] Michel JP Goldberg J. Education, Healthy Ageing and Vaccine Literacy. J Nutr Health Aging (2021) 25(5):698–701. 10.1007/s12603-021-1627-1 33949640 PMC8040006

