# Supplementary material for: Vaccine Literacy and Vaccination: A Systematic Review
Source: Int J Public Health. 2023 Feb 14;68:1605606. doi: 10.3389/ijph.2023.1605606 (PMC9970990; doi:10.3389/ijph.2023.1605606)
Supplement: Supplementary file 1 [file DataSheet1.DOCX]

**Appendix Table 1. MEDLINE Search Strategy (Shanghai, China, 2022).**

Database: MEDLINE via PubMed

Search Date:7-14-2022; searcher:DZY&ZEM

| Set # |  | Results |
| --- | --- | --- |
| 1 | ("vaccin*"[tiab] AND "literacy"[MeSH]) OR ("vaccin*"[tiab] AND "literacy"[Title/Abstract]) OR ("health literacy"[MeSH] AND "vaccin*"[tiab]) | 634 |
| 2 | "vaccines"[MeSH] OR "vaccin*"[tiab] OR "immunization"[MeSH Terms] OR "immuniz*"[tiab] OR "immunis*"[tiab] | 563,816 |
|  | 1 AND 2 | 634 |
|  | NOT (animals[mh] NOT humans[mh])  NOT (Editorial[ptyp] OR Comment[ptyp]) | 625 |

**Appendix Table 2. Embase Search Strategy (Shanghai, China, 2022).**

Database: Embase via Elsevier

Search Date:7-14-2022; searcher:DZY&ZEM

| Set # |  | Results |
| --- | --- | --- |
| 1 | (vaccin*: ti,ab,kw AND 'literacy'/exp) OR (vaccin*:ti,ab,kw AND literacy:ti,ab,kw) OR ('health literacy'/exp AND vaccin*:ti,ab,kw) | 890 |
| 2 | 'vaccine'/exp OR vaccin*: ti,ab,kw OR 'immunization'/exp OR immuniz*:ti,ab,kw OR immunis*:ti,ab,kw | 709,292 |
|  | 1 AND 2 | 890 |
|  | AND [humans]/lim | 858 |
|  | NOT 'conference abstract'/it | 744 |

**Appendix Table 3. Web of Science Search Strategy (Shanghai, China, 2022).**

Database: Web of Science Core Collection Citation Indexes ClarivateSearch Date:7-14-2022; searcher:DZY&ZEM

| Set # |  | Results |
| --- | --- | --- |
| 1 | TS= ((vaccin* AND literacy) OR ("health literacy" AND vaccin*)) | 727 |
| 2 | TS= (vaccin* OR immuniz* OR immunis*) | 403,280 |
|  | 1 AND 2 | 727 |

**Appendix Table 4. CINAHL Search Strategy (Shanghai, China, 2022).**

Database: CINAHL (Cumulative Index of Nursing and Allied Health Literature via EBSCO)

Search Date:7-14-2022; searcher:DZY&ZEM

| Set # |  | Results |
| --- | --- | --- |
| 1 | (TI vaccin* AND (MH "Literacy")) OR (AB vaccin* AND (MH "Literacy")) OR TI (vaccin* AND literacy) OR AB (vaccin* AND literacy) OR ((MH "Health Literacy") AND TI vaccin*) OR ((MH "Health Literacy") AND AB vaccin* ) | 252 |
| 2 | (MH "Vaccines+") OR (MH "Immunization+") OR TI vaccin* OR AB vaccin* OR TI immuniz* OR AB immuniz* OR TI immunis* OR AB immunis* | 94,629 |
|  | 1 AND 2 | 252 |

**Appendix Table 5. PsycINFO Search Strategy (Shanghai, China, 2022).**

Database: APA PsycINFO via EBSCO

Search Date:7-14-2022; searcher:DZY&ZEM

| Set # |  | Results |
| --- | --- | --- |
| 1 | (TI vaccin* AND DE "Literacy") OR (AB vaccin* AND DE "Literacy") OR TI (vaccin* AND literacy) OR AB (vaccin* AND literacy) OR (DE "Health Literacy" AND TI vaccin* ) OR (DE "Health Literacy" AND AB vaccin* ) | 146 |
| 2 | DE "Vaccination" OR DE "Immunization" OR TI vaccin* OR AB vaccin* OR TI immuniz* OR AB immuniz* OR TI immunis* OR AB immunis* | 10,869 |
|  | 1 AND 2 | 146 |

**Appendix Table 6. Cochrane Library Search Strategy (Shanghai, China, 2022).**

Database: Cochrane Library

Search Date:7-14-2022; searcher:DZY&ZEM

| Set # |  | Results |
| --- | --- | --- |
| 1 | MeSH descriptor: [Health Literacy] explode all trees | 469 |
| 2 | (vaccin*): ti,ab,kw | 28665 |
| 3 | 1 AND 2 | 1 |
| 4 | (vaccin* AND literacy): ti,ab,kw | 75 |
| 5 | MeSH descriptor: [Literacy] explode all trees | 56 |
| 6 | 2 AND 5 | 0 |
| 7 | 3 OR 4 OR 6 | 75 |
| 8 | (vaccin* OR immuniz* OR immunis*): ti,ab,kw | 30546 |
| 9 | 7 AND 8 | 75 |

**Appendix Table 7. General characteristics of included studies (Shanghai, China, 2022).**

| **Author, Year** | **Country** | **Study design and method and time of data collection** | **Type of Vaccination** | **Scope of Study** | **Study Sample** | **VL Areas Investigated, VL Measurement Tools** | **VL and Vaccination：Results** |
| --- | --- | --- | --- | --- | --- | --- | --- |
| Lee et al.,  2015 ^18^ | USA | Cross-sectional online survey  The 2012-2013 and 2013-2014 school years' fall semesters | HPV vaccination | To examine into factors that influence whether young Asian, American, and Pacific Islander (AAPI) and non-Latina White (NLW) women get the HPV vaccine. | This study was conducted at a public university in the Midwest (USA) and involved 2,270 women of college-going age (18–25 years): 341 AAPI and 1,929 NLW. | Based on the percentage of correctly answered responses on a five-item questionnaire that was adapted from the National Cancer Institute's vaccination recommendations, HPV-VL was determined. | HPV-VL was a significant predictor of completion of the HPV vaccine in both groups of undergraduate women. |
| Aharon et al., 2017 ^22^ | Israel | Retrospective cross-sectional telephone survey  December 2012 through March 2013 | Vaccination against Hepatitis B, Diphtheria, Tetanus, Pertussis, Measles, Rubella, and Mumps. (These are routinely given during the first year of life and constitute the core vaccination protocol) | To examine the association between parents' vaccine health literacy and their adherence to recommended child vaccines. | 731 parents of children between the ages of three and four  There are two distinct groups: Children who did not receive at least one of the three recommended childhood vaccines made up the study group. Children who have gotten all three vaccines are in the control group. | The Vaccine Health Literacy Scale was used to measure functional, communicative, and critical VL. It is a 13-item questionnaire based on the HL Questionnaire, which was made by Ishikawa. | The likelihood of parents who have highly functional, communicative, and critical VL not getting their children vaccinated is higher. Communicative VL has a substantial negative direct connection with vaccine compliance (through a significant negative indirect association). |
| Wang et al.,  2018 ^23^ | China | Cross-sectional questionnaire survey  April 2016 | Childhood Vaccinations in Domestic Production | To examine the connection between VL and vaccination-related consequences following false reports about a controversy involving locally produced children's vaccines that surfaced in China throughout 2016. | 1864 parents with children under the age of six | A 2-item questionnaire was used to figure out the functional VL, while a 3-item questionnaire made with information from the CDC and WHO was used to figure out the critical VL. | Parents with a higher VL in both understanding information about the vaccine crisis and critical thinking were more likely to trust and choose immunizations made locally. |
| Sumile et al.,  2020 ^24^ | Philippine | Cross-sectional questionnaire survey  August – November 2018 | Dengue Vaccination | To investigate the relationship of vaccine health literacy and dengue vaccine controversy awareness with vaccine acceptability | 200 mothers in rural communities of Bulaca who with children aged six months to five years or with children who have been vaccinated by the government under the immunization program of the Philippines | Functional, communicative, and critical VL was investigated using Aharon's Vaccine Health Literacy Scale with 13 items | Mothers' functional VL and critical VL revealed a significant relationship to vaccine acceptability. Communicative VL showed no significant relationship to vaccine acceptability. |
| Biasio et al.,  2021 ^26^ | Italy | Two-week online cross-sectional questionnaire survey beginning June 6, 2020 | COVID-19 Vaccination | Examine the levels of VL in the general population, how people feel about the candidates for the COVID-19 vaccine, and how and why people get vaccinated. | 885 Italian adults aged 18 years and older | The VL levels were determined by converting 12 questions from Biasio's Health Literacy about Vaccination of adult in Italian (HLVa-IT). | Perceptions of future COVID-19 vaccinations and vaccination beliefs were largely positive and substantially associated with functional and interactive-critical VL. All of the relationships between functional and interactive-critical VL scores and acceptance of current immunizations (flu and other vaccines) were significant. |
| Gendler et al.,  2021 ^25^ | Israel | Cross-sectional online questionnaire survey  June 2021 | COVID-19 Vaccination | To determine the impact of VL, perception, hesitation, and behavior on Israeli parents' intentions to vaccinate their 12–15-year-old children against COVID-19. | 520 parents of children 12-15 years old | The VL levels were evaluated using 12 items adapted from Biasio's HLVa-IT. | Parents who chose to vaccinate their children had higher mean levels of functional, interactive/critical, and total VL ratings, as well as a more positive impression of the vaccine and less vaccine hesitation. |
| Gusar et al.,  2021 ^27^ | Croatian | Cross-sectional online  questionnaire survey  15 to 31 January 2021 | COVID-19 Vaccination | Prior to immunization, evaluate the prevalence of COVID-19 VL in the general adult population of Croatia. The aims were to test for socio-demographic disparities and assess views and attitudes regarding vaccination against COVID-19 considering the degree of VL against COVID-19. | 1227 non-COVID-19 vaccinated adults over 18 years of age | The functional and interactive-critical VL was investigated using Biasio's HLVa-IT, with 14 items. | Participants with significantly higher levels of functional and interactive-critical COVID-19 VL responded more frequently and positively to questions regarding the production of safe and effective vaccines, their personal reactions to vaccination, the possibility of vaccinating the entire population, and the necessity of vaccinating children. |
| Khiari et al.,  2021 ^36^ | Tunisia | Cross-sectional  questionnaire survey  February 2021 | COVID-19 Vaccination | To ascertain the acceptance rate of the COVID-19 vaccination among cancer patients at the Salah Azaiez Institute (SAI) and to pinpoint the contributing factors. | 200 cancer patients above 18 years of age, who were admitted to the hospital for treatment during the month of February 2021 | 12 items from Biasio's HLVa-IT were modified to assess functional and interactive-critical VL. | The interactive VL score was very associated with the acceptance of the COVID-19 vaccination. However, no significant association was found between the acceptance to get the vaccine and the functional VL score |
| Krishnamurthy et al.,  2021 ^38^ | Barbados | Cross-sectional online  questionnaire survey  February 14 and 27, 2021 | COVID-19 Vaccination | To identify the perceptions and attitudes of HCPs at the Queen Elizabeth Hospital (QEH) in Barbados about the acceptability of the COVID-19 vaccine. | The 343 HCPs currently working at QEH include physicians, nurses, pharmacists, physical therapists, radiographers, laboratory technicians/technicians, and others. | In accordance with Nutbeam's criteria, the interactive-critical VL were evaluated by adopting six items from Biasio's HLVa-IT. | Those who stated they would take the vaccine had a mean VL score that was greater than that of those who were not prepared to take the vaccine immediately. |
| Nath et al.,  2021 ^28^ | Bangladesh | Cross-sectional online  questionnaire survey  February to September 2021* | COVID-19 Vaccination | To investigate the relationship of vaccination hesitancy, eHealth literacy, and VL with young persons' desire to receive the COVID-19 vaccine in Bangladesh. | 343 young adults aged 18-30 years old | Adapting 12 items from Biasio's HLVa-IT, the functional and interactive-critical VL were measured. | No significant correlation between VL and COVID-19 vaccine uptake intention. |
| Yadete et al.,  2021 ^33^ | USA | Cross-sectional online  questionnaire survey  14 July to 19 July 2021 | Booster dose of COVID-19 vaccination | To evaluate the acceptance of booster doses and associated determinants among Americans who are vaccine eligible. | 2317 present U.S. citizens who are at least 18 years old and can understand and give informed consent in English. | The VL levels were assessed by using Biasio's HLVa-IT, with 14 items | The average VL scores for functional, interactive (communicative), and critical literacy were much higher in the group that was open to getting boosters than in the group that wasn't. |
| Achrekar et  al.,  2022 ^34^ | India | Cross-sectional online  questionnaire survey  13 December 2021 to  10 February 2022 | Booster dose of COVID-19 vaccination | To examine the acceptability of booster doses and associated predictors in the Indian population. | 687 Indian citizens over the age of 18 | Functional, communicative, and critical VL were assessed by using Biasio's HLVa-IT, with 14 items | The group that wasn't hesitant had much higher average functional, communicative, critical, and total VL scores than the group that was hesitant. |
| Alshehry et al.,  2022 ^17^ | Saudi Arabia | Cross-sectional online  questionnaire survey  November 26 to December 31, 2020 | COVID-19 Vaccination | To examine the predictors of nursing students’ intention to vaccinate against COVID‐19 | 1170 nursing students between the ages of 18 and 37 years in ten public universities | Twelve items from Biasio's HLVa-IT were modified to measure functional and interactive-critical VL. | High levels of interactive‐critical COVID‐19 VL were significant predictors of intentions to vaccinate against COVID‐19 among nursing students |
| Batra et al.,  2022 ^35^ | USA | Cross-sectional online  questionnaire survey  October 2021 | Booster dose of COVID-19 vaccination | To research the effects of hesitancy, confidence, literacy, and the multi-theory model (MTM) components on the uptake of COVID-19 booster. | 501 adults aged 18 and over | The VL levels were assessed by using Biasio's HLVa-IT, with 14 items. | The hesitant group had statistically significantly lower mean scores for functional, interactive, or communicative, critical, and total VL than the non-hesitant group. |
| Carter et al.,  2022 ^40^ | Australia | Cross-sectional online  questionnaire survey  1 June to 24 July 2021 | COVID-19 Vaccination | To examine the factors affecting COVID-19 vaccine uptake in younger Australian women living in rural and regional communities | 90 younger Australian women aged between 30–44 | By adopting 12 items from Biasio's HLVa-IT, the functional and interactive-critical VL were measured. | The intention to receive a COVID-19 vaccination was not significantly associated with VL. |
| Correa-Rodríguez et al.,  2022 ^37^ | Spain | Cross-sectional online  questionnaire survey  May 8 to June 8, 2021 | COVID-19 Vaccination | 1. To evaluate for the first time VL skills in a population of patients with systemic autoimmune diseases. 2. To examine the potential associations between VL skills and sociodemographic characteristics. 3. To analyze the relationships between attitudes, perceptions, and beliefs about current vaccinations and VL skills and sociodemographic characteristics. | 319 adults with systemic autoimmune disorders were evaluated. | The VL levels were assessed by using Biasio's HLVa-IT, with 14 items | VL is associated with positive attitudes and perceptions of the COVID-19 vaccine, but not with current vaccination behaviour. Interactive critical VL was more likely to be associated with positive beliefs about COVID-19 vaccination |
| Engelbrecht et al.,  2022 ^29^ | South Africa | Cross-sectional online  questionnaire survey  September 2021 | COVID-19 Vaccination | To determine the levels of VL in the adult population of South Africa and to find the causes of low VL. | 10466 adults aged 18 and over | 12 items from Biasio's HLVa-IT were modified to assess functional and interactive-critical VL. | Individuals who had limited functional and interactive-critical VL were more likely to not have received the COVID-19 vaccination. Having limited vaccine literacy is indicated by a score of 2.50. |
| Engelbrecht et al.,  2022 ^30^ | South Africa | Cross-sectional online  questionnaire survey  September 2021 | COVID-19 Vaccination | To determine the causes of vaccination hesitancy and non-uptake of the COVID-19 vaccine. | 10466 adults aged 18 and over | 12 items from Biasio's HLVa-IT were modified to assess functional and interactive-critical VL. | The completion of the COVID-19 vaccination was significantly predicted by the functional and interactive-critical VL.  Compared to people with lower levels of interactive-critical VL, people with restricted levels of interactive-critical VL were about twice as likely to be vaccine-hesitant. |
| Maneesriwongul et al.,  2022 ^31^ | Thailand | Cross-sectional online  questionnaire survey  May 2021 | COVID-19 Vaccination | To translate and test the  psychometric properties of the Thai COVID-19 VL  Scale for its use in Thailand. | 1002 adults aged 18 years and older | The Thai COVID-19 Vaccine Literacy Scale with 12 items, translated by Biasio's HLVa-IT, was used to assess functional and interactive-critical VL. | Participants who were vaccinated against COVID-19 had significantly higher VL scores than those who intended and who were not sure/ not willing to get vaccination |
| Omidvar et al.,  2022 ^32^ | Iranian | Cross-sectional online  questionnaire survey  May 2021 | COVID-19 Vaccination | To ascertain the level of acceptance of COVID-19 vaccine in the Iranian community and the factors influencing it. | 1564 Iranians over the age of 18 who are literate in reading and writing, have access to smartphones, laptops, or tablets, and use social media. | By adopting 12 items from Biasio's HLVa-IT, the functional and interactive-critical VL were measured. | VL were indicators of COVID-19 vaccination uptake. |
| Siewchaisakul et al.,  2022 ^39^ | Thailand | Cross-sectional online  questionnaire survey  1 to 15 October，2021 | COVID-19 Vaccination | To investigate the consequences of VL, vaccine fear (VF) and vaccine hesitancy (VH) on acceptance of COVID-19 vaccine among village health volunteers (VHVs). | 5312 VHVs aged over 18 and registered in the mobile application SMART VHV. | Functional, communicative, and critical VL were assessed by adapting 12 questions from Biasio's HLVa-IT. | The VL was insignificantly associated with increased COVID-19 vaccination. |

*Nath et al. didn’t specify the date of data collection; we speculated through the revision and publication time of the literature.

**Appendix Table 8. Quality assessment of 21 included studies (Shanghai, China, 2022).**

| **ITEM (description)** | | **1** | **2** | **3** | **4** | **5** | **6** | **7** | **8** | **9** | **10** | **11** | **12** | **13** | **14** | **15** | **16** | **17** | **18** | **19** | **20** | **21** |
| --- | --- | --- | --- | --- | --- | --- | --- | --- | --- | --- | --- | --- | --- | --- | --- | --- | --- | --- | --- | --- | --- | --- |
| **Title and abstract** | | 1 | 1 | 1 | 1 | 1 | 1 | 1 | 1 | 1 | 1 | 1 | 1 | 1 | 1 | 1 | 1 | 1 | 1 | 1 | 1 | 1 |
| **Introduction** | **Background/rationale** | 1 | 1 | 1 | 1 | 1 | 1 | 1 | 1 | 1 | 1 | 1 | 1 | 1 | 1 | 1 | 1 | 1 | 1 | 1 | 1 | 1 |
|  | **Objectives** | 1 | 1 | 1 | 1 | 1 | 1 | 1 | 1 | 1 | 1 | 1 | 1 | 1 | 1 | 1 | 1 | 1 | 1 | 1 | 1 | 1 |
| **Methods** | **Study design** | 1 | 1 | 1 | 1 | 1 | 1 | 1 | 1 | 1 | 1 | 1 | 1 | 1 | 1 | 1 | 1 | 1 | 1 | 1 | 1 | 1 |
|  | **Setting** | 1 | 1 | 1 | 1 | 1 | 1 | 1 | 1 | 1 | 0 | 1 | 1 | 1 | 1 | 1 | 1 | 1 | 1 | 1 | 1 | 1 |
|  | **Participants** | 1 | 1 | 1 | 1 | 1 | 1 | 1 | 1 | 1 | 1 | 1 | 0 | 1 | 1 | 1 | 1 | 1 | 1 | 1 | 1 | 1 |
|  | **Variables** | 1 | 1 | 1 | 1 | 1 | 1 | 0 | 0 | 0 | 1 | 0 | 0 | 1 | 1 | 1 | 1 | 1 | 1 | 1 | 1 | 1 |
|  | **Data sources/measurement** | 1 | 1 | 1 | 1 | 1 | 1 | 0 | 1 | 0 | 1 | 0 | 0 | 1 | 1 | 1 | 1 | 1 | 1 | 1 | 1 | 1 |
|  | **Bias** | 0 | 0 | 0 | 0 | 0 | 0 | 0 | 0 | 0 | 1 | 1 | 0 | 0 | 0 | 0 | 0 | 0 | 0 | 0 | 0 | 0 |
|  | **Study size** | 1 | 0 | 0 | 0 | 0 | 1 | 0 | 0 | 0 | 1 | 1 | 1 | 0 | 1 | 1 | 0 | 0 | 0 | 0 | 1 | 0 |
|  | **Quantitative variables** | 1 | 1 | 1 | 0 | 1 | 1 | 1 | 1 | 0 | 1 | 0 | 0 | 1 | 1 | 1 | 0 | 1 | 1 | 1 | 1 | 1 |
|  | **Statistical methods** | 1 | 1 | 1 | 1 | 1 | 1 | 1 | 1 | 1 | 1 | 1 | 1 | 1 | 1 | 1 | 1 | 1 | 1 | 1 | 0 | 1 |
| **Results** | **participants** | 1 | 1 | 1 | 1 | 1 | 1 | 1 | 1 | 1 | 0 | 1 | 1 | 1 | 0 | 0 | 1 | 1 | 1 | 1 | 1 | 1 |
|  | **Descriptive data** | 1 | 1 | 1 | 1 | 1 | 1 | 1 | 1 | 1 | 1 | 1 | 1 | 1 | 1 | 1 | 1 | 1 | 1 | 1 | 1 | 1 |
|  | **Outcome data** | 1 | 1 | 1 | 1 | 1 | 1 | 1 | 1 | 1 | 1 | 1 | 1 | 1 | 1 | 1 | 1 | 1 | 1 | 1 | 1 | 1 |
|  | **Main results** | 1 | 1 | 1 | 1 | 1 | 1 | 1 | 1 | 1 | 1 | 1 | 1 | 1 | 1 | 1 | 1 | 1 | 1 | 1 | 1 | 1 |
|  | **Other analyses** | 0 | 1 | 0 | 0 | 0 | 1 | 0 | 0 | 0 | 1 | 0 | 0 | 0 | 1 | 0 | 0 | 0 | 0 | 0 | 0 | 1 |
| **Discussion** | **Key results** | 1 | 1 | 1 | 1 | 1 | 1 | 1 | 1 | 1 | 1 | 1 | 1 | 1 | 1 | 1 | 1 | 1 | 1 | 1 | 1 | 1 |
|  | **Limitations** | 1 | 1 | 1 | 0 | 1 | 1 | 1 | 0 | 1 | 1 | 1 | 1 | 1 | 1 | 1 | 1 | 1 | 1 | 1 | 1 | 1 |
|  | **Interpretation** | 1 | 1 | 1 | 1 | 1 | 1 | 1 | 1 | 1 | 1 | 1 | 1 | 1 | 1 | 1 | 1 | 1 | 1 | 1 | 1 | 1 |
|  | **Generalizability** | 1 | 1 | 1 | 1 | 1 | 1 | 1 | 1 | 1 | 1 | 1 | 1 | 1 | 1 | 1 | 1 | 1 | 1 | 1 | 1 | 1 |
| **Other information** | **Funding** | 1 | 1 | 1 | 1 | 0 | 1 | 1 | 0 | 0 | 1 | 1 | 1 | 0 | 1 | 1 | 1 | 1 | 1 | 0 | 1 | 1 |
| **Score** | | 20 | 20 | 19 | 17 | 18 | 21 | 17 | 16 | 15 | 20 | 18 | 16 | 18 | 20 | 19 | 18 | 19 | 19 | 18 | 19 | 20 |
